# Early cognitive assessment in premature infants: the discriminatory value of eye-tracking vs. Bayley Scales

**DOI:** 10.3389/fpsyg.2024.1384486

**Published:** 2024-06-18

**Authors:** Maria Kaltsa, Evgenia Babacheva, Georgia Fotiadou, Evanthia Goutsiou, Katerina Kantziou, Katerina Nicolaidis, Vasiliki Soubasi

**Affiliations:** ^1^Language Development Lab, School of English, Department of Theoretical and Applied Linguistics, Aristotle University of Thessaloniki, Thessaloniki, Greece; ^2^2^nd^ Department of Neonatology and NICU, School of Medicine, General Hospital of Papageorgiou, Aristotle University of Thessaloniki, Thessaloniki, Greece; ^3^LingLab, School of Philology, Department of Linguistics, Aristotle University of Thessaloniki, Thessaloniki, Greece; ^4^1^st^ Neonatal Department and NICU, Hippokration General Hospital, Aristotle University of Thessaloniki, Thessaloniki, Greece; ^5^Phonetics Laboratory, School of English, Department of Theoretical and Applied Linguistics, Aristotle University of Thessaloniki, Thessaloniki, Greece

**Keywords:** eye-tracking, cognitive screening, Bayley Scales, prematurity, neurodevelopmental delays, assessment

## Abstract

**Introduction:**

The testing of visuocognitive development in preterm infants shows strong interactions between perinatal characteristics and cognition, learning and overall neurodevelopment evolution. The assessment of anticipatory gaze data of object-location bindings via eye-tracking can predict the neurodevelopment of preterm infants at the age of 3 years; little is known, however, about the early cognitive function and its assessment methods during the first year of life.

**Methods:**

The current study presents data from a novel assessment tool, a Delayed Match Retrieval (DMR) paradigm via eye-tracking was used to measure visual working memory (VWM) and attention skills. The eye-tracking task that was designed to measure infants’ ability to actively localize objects and to make online predictions of object-location bindings. 63 infants participated in the study, 39 preterm infants and 24 healthy full term infants – at a corrected age of 8–9 months for premature infants and similar chronological age for full term infants. Infants were also administered the Bayley Scales of Infant and Toddler Development.

**Results:**

The analysis of the Bayley scores showed no significant difference between the two groups while the eye-tracking data showed a significant group effect on all measurements. Moreover, preterm infants’ VWM performance was significantly lower than full term’s. Birth weight affected the gaze time on all Areas Of Interest (AOIs), overall VWM performance and the scores at the Cognitive Bayley subscale. Furthermore, preterm infants with fetal growth restriction (FGR) showed significant performance effects in the eye-tracking measurements but not on their Bayley scores verifying the high discriminatory value of the eye gaze data.

**Conclusion:**

Visual working memory and attention as measured via eye-tracking is a non-intrusive, painless, short duration procedure (approx. 4-min) was found to be a significant tool for identifying prematurity and FGR effects on the development of cognition during the first year of life. Bayley Scales alone may not pick up these deficits. Identifying tools for early neurodevelopmental assessments and cognitive function is important in order to enable earlier support and intervention in the vulnerable group of premature infants, given the associations between foundational executive functional skills and later cognitive and academic ability.

## 1 Introduction

Prematurity is a global problem affecting directly the community and many scientific areas due to its association with many short and long term neonatal complications (Patel, 2016; Humberg et al., 2020; Smyrni et al., 2021). Α premature delivery can be characterized as a one syndrome, with many different causes leading to this and is defined as birth before 37 completed weeks of gestation (Romero et al., 2014). It is estimated to have affected 13.4 million babies born in 2020 (Ohuma et al., 2023). These infants experience significant morbidity and mortality in the newborn period and may face a number of health-related problems, such as motor delay and cerebral palsy, lower cognition along with other behavioral issues. There is significant evidence in the literature for the association between prematurity with developmental disorders, which is inversely proportional to gestational age (GA hereafter) [Stevenson et al., 1988; Marenne, 1989; Censullo, 1994; Mellier et al., 1999; Buck et al., 2000; Magny and Rigourd, 2003; Jarjour, 2015; Pascal et al., 2018; Lowe et al., 2020; Beunders et al., 2021 – for discussion of sub-categories of preterms see Goldenberg et al. (2008) and Raju et al. (2006)] and as such it is critical that we identify cognitive delays early on so as to introduce appropriate interventions.

Apart from GA, another parameter to be considered when examining (a) typical neurodevelopment in infants is birth weight and in particular, very low birth weight (VLBW less than 1,500 gr.), otherwise referred to as small for gestational age (SGA: defined as birthweight <10th percentile) (McCowan and Horgan, 2009). SGA infants may or may not be with fetal growth restriction (FGR) (Sacchi et al., 2020) which is associated to maternal, placental, fetal origin or other environmental factors; these perinatal factors appear to affect brain development and consequently skills such as learning, memory and overall cognitive function (see Algarín et al., 2003; Atkinson and Braddick, 2007; Fransson et al., 2007; Amin et al., 2013; Malhotra et al., 2019; Castro Conde et al., 2020; Melamed et al., 2021 among others). Recent research suggests that SGA infants in particular appear to be at higher risk of neurodevelopmental problems (Bickle Graz et al., 2015; Meher et al., 2015; Larsen et al., 2022; Naz et al., 2023) with the possibility, though, of positive neurodevelopmental outcomes in the case of early postnatal growth (Taine et al., 2018). Brain development overall and that of specific neural structures such as the hippocampus is regulated by fetal-neonatal characteristics and have a direct impact on the development of recognition, memory and learning. Earlier studies assessed auditory recognition memory in infants via event-related potentials (ERP) and identified impairments that related to the development of the hippocampus (deRegnier et al., 2000; Siddappa et al., 2004; Geng et al., 2015) offering further support that the perinatal period and the first year of life are key in neurodevelopmental terms.

Neurodevelopment is typically monitored via clinical assessment and the administration of standardized tests such as the Bayley Scales of Infant and Toddler Development (Bayley) to infants and children from birth up to the age of 3;6 years. Bayley-III (3rd edition) is a widely used and highly reliable tool (Bayley – III, 2006a; Yue et al., 2019; Del Rosario et al., 2021) that assesses cognition, receptive and expressive communication, fine and gross motor skills, socioemotional and adaptive behavior. The administration of such tools has assisted in the early detection of developmental delays in infants born prematurely (Aylward, 2002). Most research has focused on the role of neonatal characteristics of premature infants at the corrected age (CA) of 2 to 2;6 years of age (Duncan et al., 2012) and showed that cognitive, language, and motor neurodevelopment, as measured via the Bayley scales, is indeed delayed (Greene et al., 2012). This finding was also replicated by Velikos et al. (2015) who tested premature infants at the CA of 12 months suggesting that developmental delays can be identified earlier on. Little is known, however, with regard to the sensitivity of the tool to identify such delays due to prematurity earlier on within the first year of life.

Apart from standardized tools that provide an overview of an infant’s performance, there has been extensive work on cognitive development looking at a variety of subdomains and possible measurements throughout infancy and childhood. Cognitive development theories attempt to describe information processing at different developmental stages examining how children identify, use and store information with critical milestones being related to changes in the amount of information that the child can sort, classify and use (Gathercole, 1999; Baddeley, 2003; Gathercole et al., 2003; Baddeley, 2012; Cowan, 2016 among others). Working memory (WM) is a critical component of executive functions which assist (non)verbal comprehension, learning and building knowledge (Cowan, 2016). Often its capacity is treated as a maturation index for developmental studies. The focus of research on WM growth in infancy as opposed to childhood has provided a lot of new information on the skills of infants such as their understanding of object/event properties (Spelke et al., 1992), enumeration of small numbers of objects (Wynn, 1996), transitive inferences (Mou et al., 2014), and false beliefs (Choi and Luo, 2015). Infant studies on WM suggest that typically developing 6-month-old infants can respond well in tasks with only a single item to be remembered (Simmering, 2012; Oakes et al., 2013; Kibbe, 2015; Zosh and Feigenson, 2015), while infants older than 8 months appear to have a capacity of about 3 items (Kibbe and Leslie, 2013). It is crucial to underscore though that the uniqueness of the objects employed in such tasks may have an effect in the number of items they can remember (Oakes et al., 2013). Additionally, since these types of studies measure looking responses, it is often questioned how automatic or deliberate that process is (Zosh and Feigenson, 2015).

Considering the difficulties of measuring WM growth, an examination of the development of visual attention during the first year of life could potentially be more informative. Attention is typically treated as maintaining an alert state, orienting to stimuli, and regulating the response to that sensory event – alerting, orienting, and executive attention (Posner and Petersen, 1990; Posner and Fan, 2008; Petersen and Posner, 2012; Posner, 2012; Swingler et al., 2015; Oakes, 2023). The infants’ visual attention increases dramatically during the second half of the first year of life and it has been shown to be a significant predictor of childhood cognitive functioning and associated outcomes (Posner and Fan, 2008; Colombo et al., 2010; Posner, 2012). Specifically, from birth to 8–10 weeks of age looking duration increases and attention engagement abilities start building; the looking duration of infants between the ages of 3 to 6 months declines as information processing improves with shorter durations of attention engagement required to process a stimulus; lastly, 7-month-old and older infants increase response to more complex stimuli (Swingler et al., 2015).

With regard to the emergence of executive attention, Cuevas and Bell (2014) tested infant attention and early childhood executive function via longitudinal data of 5-month-olds reexamined at 24, 36, and 48 months of age. Their analysis suggests that infants with more efficient information processors showed higher executive function throughout early childhood. Moreover, two studies, one with infants in Italy and one with infants in Japan, showed that 8-month-old typically developing infants were faster to look at targets that appeared at uncued locations on the same object than at uncued locations on a different object (Bulf and Valenza, 2013; Tsurumi et al., 2018). Their evidence suggests that (a) visual objects can operate as units of attention for infants by that age and (b) object-based attention and spatial orienting develop across infancy. Kaldy et al. (2016) tested 8- and 10-month-old infants’ visual working memory (VWM) for object-location binding via eye-tracking using a Delayed Match Retrieval (DMR) task using a memory game and reported that even though the performance of 8-month-old infants was at chance, 10-month-old infants performed significantly above chance, showing that their VWM could hold object-location information. The data of these studies concern full term infants and provide useful information in the domain of cognitive development; there is very limited research though on preterm infants, whether and how they may differ to full term ones as well as the discriminatory value of such online tasks in relation to broader offline neurodevelopmental assessment tools.

Atkinson and Braddick (2012) do highlight that neurocognitive impairment is frequent for births before 32 weeks’ gestation, since damage in developing oligodendrocytes due to ischaemic events, and/or early infections lead to white matter injury (du Plessis and Volpe, 2002). Additionally, there is evidence that attention problems in childhood can be linked even to moderate preterm birth (for reviews see van de Weijer-Bergsma et al., 2008; Mulder et al., 2009). Few studies have looked specifically into the visuospatial and object orienting skills of preterm infants. Kaul et al. (2016) tested very preterm infants at two-time intervals; at 4 months-old CA and at 2;6–3;6 years old and found that (a) gaze gain was related to GA and prematurity, and (b) the ability to visually track a moving object at 4 months can predict neurodevelopment at 3 years of age of very preterm infants. Moreover, Ross-Sheehy et al. (2017) developed the Infant Orienting With Attention (IOWA) task to measure the infants’ visual attention using a target-object (e.g., an umbrella, a peach, a cow) in several positions on the screen, along with peripheral cues (black dots) and an engaging image as a fixation stimulus. The analysis of 5- and 10-month-old preterm and full-term infant data showed that in typical development 5-month-old infants’ visual spatial attention is highly accurate, while preterm infants exhibit attentional deficits quite early on. Note that both the Kaul et al. (2016) and the Ross-Sheehy et al. (2017) studies developed tasks that do not require any working memory involvement while Kaldy et al. (2016) does, hence the differences in the ages of infants participating in the studies and their respective outcomes.

Considering the findings in visuocognitive development in preterm infants, we can hypothesize that there are strong interactions between perinatal characteristics and neurodevelopment in infants. Both gross measures such as those from standardized testing and experimental designs that exploit the assessment of (anticipatory) gaze data of object-location bindings via newer methodologies such as eye-tracking are shown to be possible predictors for the neurodevelopment of preterm infants. The goal of the present study is to evaluate the eye-tracking methodology, which offers a novel assessment tool with unbiased, objective and quantifiable data, and evaluate its discriminatory value in comparison to that of off-line testing, and in particular that of Bayley-III. Specifically, we aim at testing the effects of prematurity in visual working memory and attention via eye-tracking and in performance in the Bayley Scales in 8- to 9-month-old infants so as to compare the two tools and identify the one that best detects neurodevelopmental delays.

## 2 Materials and methods

### 2.1 Participants

A total of 72 8- to 9-month-old infants participated in the present research. Nine were excluded from further analyses due to one of the following exclusion criteria; high proportion of eye-movement data was missing (>60%) (N: 3), technical error (N: 2), child’s lack of concentration (N: 2) or other administration error (N: 2). Two groups were formed; a group of preterm infants (N: 39) and a group of healthy full-term infants (N: 24) – see Table 1. For recruitment we collaborated with a large academic medical center, specifically the Papageorgiou General Hospital of Thessaloniki in Northern Greece. The study was approved by the Scientific Council and the Ethics Committee of Papageorgiou General Hospital of Thessaloniki, Greece [Ethics Approval N: Δ3β/28893 | D3b/28893] and written informed consent was obtained from parents/guardians of the infants during recruitment. Preterm infants were cared for in the Neonatal Intensive Care Unit (NICU) of the hospital, while full-term infants were recruited primarily via the hospital but also from various private medical settings using birth records in the same area (Thessaloniki, Northern Greece). Perinatal characteristics were collected including Gestational Age (GA), Birth Weight (BW), Small for Gestational Age (SGA), Fetal Growth Restriction (FGR), sex, Corrected Age for preterm infants (CA)^1^ along with a severity index for preterm infants (5-point scale: 0 = no issues, 1 = mild, 2 = moderate, 3 = moderate severe, 4 = severe). GA was estimated using last menstrual period, CA for preterm infants was calculated by subtracting the number of weeks born before 40 weeks of gestation from the chronological age – relevant info per group and between group comparisons where applicable are provided in Table 1. Note that the two groups differed significantly only with regard to GA and BW. Moreover, all participants underwent an ophthalmological examination. We excluded preterm infants with retinopathy of prematurity and other ophthalmological neonatal diseases, as well as premature infants with pathological findings on neuroimaging.

**Table 1:** Perinatal data per group and between group significant/non-significant differences.

	Preterm infants	Full term infants	*p*
*N*	39	24	
Sex	23 M, 16F	17M, 7F	NS
Birth weight – BW (grams)	1884 ± 684	2,898 ± 390	<0.001
Small for gestational age – SGA	8	NA	
Fetal growth restriction – FGR	13	NA	
Gestational age – GA (weeks)	32.7 ± 3.3	38.7 ± 1.5	<0.001
Corrected age – CA (months)	9.01 ± 1.8	8.1 ± 2.4	NS
Severity index	0.94 ± 0.88	NA	

Follow-up was considered routine clinical care for the preterm infants, yet all participating infants were clinically assessed. For the purposes of the study two separate sessions were additionally scheduled. A fixed procedure for test administration resulted in all infants being administered the Bayley Scales of Infant and Toddler Development – third edition (Bayley-III) first, followed by the Delayed Match Retrieval (DMR) paradigm eye-tracking task that we developed to measure visual working memory (VWM). This procedure was utilized in order to enhance the possibility that one test perform a preparation function for the other in a biased way. The Bayley-III was administered by a single examiner and the DMR eye-tracking task by another. The tools employed are outlined in detail in the following sections (see Sections 2.2 and 2.3).

### 2.2 Bayley Scales of infant and toddler development – 3rd edition

For the developmental assessment of the infants participating in the study we employed the Greek adaptation of the Bayley Scales of Infant and Toddler Development (3rd edition) (Velikos et al., 2015). The tool measures the development of infants 1 to 42 months of age in five domains: cognition, language, motor, social–emotional and adaptive behavior. The tool was initially developed for English-speaking populations but it has been adapted in a number of different languages/cultures (for Greek see Velikos et al., 2015; for Dutch see Steenis et al., 2015; for Ethiopian see Hanlon et al., 2016; for Persian see Azari et al., 2017; for Nepal Bhasa and Nepali see Ranjitkar et al., 2018; for Mandarin see Hua et al., 2019; for Kenyan see McHenry et al., 2021; for Russian see Pavlova et al., 2022 among many others). Note though that the adaptation and psychometric validation of the socioemotional and adaptive behavior domains in different language/culture contexts is not always attainable hence most prior research focuses on three scales: the cognitive scale, the language scale and the motor scale. Hence, for the needs of our research, we used the norm referenced subscale scores for three of these indices, i.e., the Cognitive, Language and Motor indices (Bayley – III, 2006b). The *Cognitive* scale assesses sensorimotor development, exploration and manipulation, object relatedness, concept formation, memory, and simple problem solving. The *Language* scale includes two subtests, the Receptive Communication and the Expressive Communication components; the former assesses word comprehension along with the child’s ability to respond appropriately to words and requests and the latter measures preverbal communication, lexical and syntactic development. Finally, the *Motor* scale also consists of two subtests; the Fine Motor component evaluates tasks such as grasping, perceptual-motor integration, motor planning, and speed and the Gross Motor component capacities such as sitting, standing, locomotion, and balance – for detailed description see Bayley – III (2006a), Velikos et al. (2015) among others. For the administration of Bayley-III, infants were comfortably seated in a noiseless room with only the presence of the examiner. The task duration was approximately 20 min.

### 2.3 Delayed match retrieval paradigm via eye-tracking

A Delayed Match Retrieval (DMR) paradigm via eye-tracking was developed to measure the visual working memory (VWM) skills of infants. Specifically, the eye-tracking task was designed to measure the infants’ ability to actively localize objects and to make online predictions of object-location bindings. The design was adapted from the Kaldy et al. (2016) DMR paradigm; we opted out of flying effects and multiple sound cues for the presentation of the objects to avoid any interference in the visual attention and memory data collected. Additionally, with regard to the stimuli shapes, we utilized combinations of the standardized Lea Symbols (Hyvärinen et al., 1980) which are of similar processing complexity ensuring that there is no disturbing visual information relating to the objects used in testing. Lea Symbols are optotypes recommended for clinical screening of visual acuity by the International Council of Ophthalmology (1984) and World Health Organization (2003); the optotypes are four outlining an *apple*, a *pentagon/house*, a *square*, and a *circle*. Moreover, the use of Lea symbols in black and white format allowed us to ensure that infants’ gaze is not conditioned by highly preferred colors (Bornstein, 1975; Adams, 1987; Zemach et al., 2007).

For the development and administration of the eye-tracking DRM task, we used Tobii T120. The task had three phases: Calibration Phase, Familiarization Phase and Testing Phase. The Calibration Phase is a procedure by which the features of an infant’s eyes are estimated for accurate gaze point calculation. The participant is presented with one target, a red dot, that appears at five points on the screen and the tracker collects data about the infant’s eyes and their gaze to that target.

Next, in the Familiarization Phase the infant is acquainted with the four Lea optotypes and the Areas of Interest (AOIs) on the 17-inch screen that those optotypes appear. For the presentation of the shapes on screen, we divided the screen into three symmetrical AOIs – top right, top left and bottom center – squaring 2 cm around each object. The Familiarization Phase includes four training trials, during which the infant sees a blank white slide for 1 s, a second slide with the optotype appearing in one of the three AOIs for 2.5 s; next, another blank slide for 1 s and a fourth slide with the symbol reappearing in another AOI for 4 s. Finally, in the fifth slide the matching pair appears along with a simultaneous wind chimes melody for 1 s. As soon as the infant completes the Calibration and Familiarization phases, the Testing Phase initiates.

**Figure 1:**
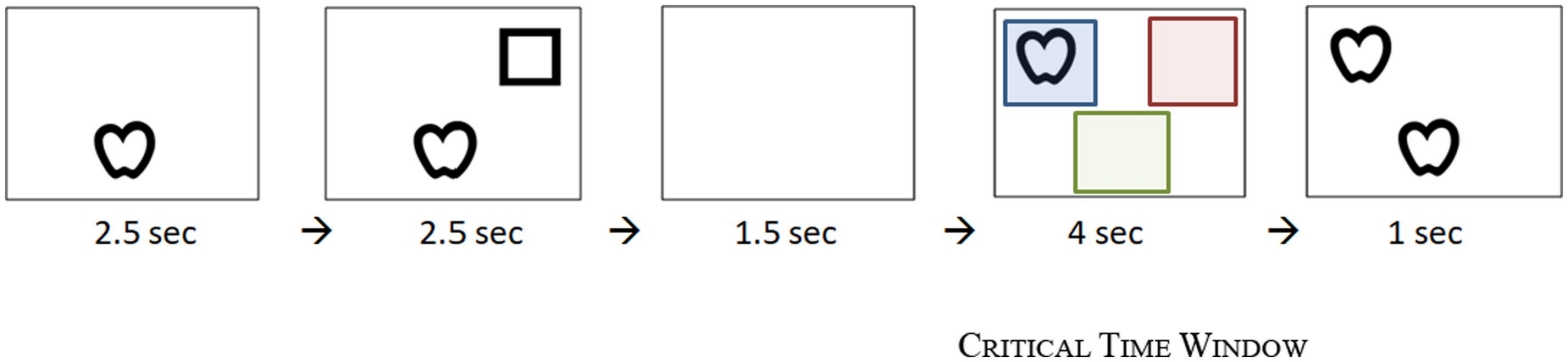
Example of series of pictures displayed in the visual-world paradigm.

During the Testing Phase (paradigm illustrated in Figure 1) the infant looks at two sets of 14 experimental trials. The second set includes the same experimental items as the first set in a reverse order to deal with order effects due to fatigue or lack of concentration. Specifically, we created two lists; in one list the order of items was introduced from 1 to 14 and then from 14 to 1 and in the second list participants were first introduced the second (reverse) order, i.e., they first saw items 14 to 1 and then items 1 to 14. Half of the participants saw the first list and the other half saw the second list. Each of the testing trials includes five stimuli slides similar to the ones of the Familiarization Phase; the first two slides appear on the screen for 2.5 s each introducing the objects that constitute a pair.

We only included two pairs of symbols throughout the test – following Hartshorne (2008), Makovski and Jiang (2008), and Kaldy et al. (2016), namely the pairs *square-apple* and *house-circle*, to decrease processing load and introduce proactive interference. In the first slide one of the four Lea optotypes (the *apple* in Figure 1) is introduced, located either on the top left or right side of the screen, or on the bottom center. In the second slide, the first object remains on the screen and another one is added (the *square* in Figure 1), in one of the other two positions. Next, there is a white slide, used to examine the direction of anticipatory looks among participants which remains on the screen for 1.5 s. In the following slide, the critical time window (TW), one of the two previous shapes reappears on the screen (either the *square* or the *apple*, as in Figure 1) in the third position; the slide remains on screen for 4 s. At this TW, we expect that the infant will gaze back to the initial position, looking for the matching shape. The trial ends with the simultaneous presentation of the pair of identical objects and a wind chimes melody; this fifth slide remains on screen for 1 s.

In between the experimental trials, a green duck (provided by Clip Art) making a quack sound appears to signal the sequence of items and regain infant’s interest at the center of the screen. For the total trials across participants, we used two versions with half of the participants seeing the reverse order. Note also that the order of item presentation was counterbalanced throughout the experiment with regard to pairs, matching objects and the position of Lea symbols across the three AOIs. With regard to data extraction, we register the fixation duration sum in the three preselected AOIs during this TW; specifically, fixation time on the object (blue square, as depicted in Figure 1), the matching pair (green square in Figure 1) and the area of the other object (red square in Figure 1).

For this eye-tracking task, infants sit on their care giver’s lap,^2^ in a distance of approximately 60 cm from the eye-tracker’s screen. The task is administered in a sound treated room (part of the Language Development and Phonetics Laboratories of the Aristotle University of Thessaloniki) with controlled light conditions; caregivers wear dark shaded sunglasses and are asked not to interact with their children during the testing procedure. The total task duration was approximately four minutes with no drop of commitment observed.^3^

### 2.4 Data analysis

For the statistical analysis of the Bayley-III and DMR eye-tracking data, we used the ΙΒΜ SPSS Statistics Software v. 28 (IBM Corp. Released 2021. IBM SPSS Statistics for Windows, Version 28.0. Armonk, NY: IBM Corp.); statistical significance was set to two-tailed *p*-value <0.05. Firstly, we performed analysis of variance (one-way ANOVA) to compare the effect of Prematurity (Preterm vs. Full term infants) on Cognitive, Language and Motor indices of the Bayley-III task and on Visual Attention (VA) and Visual Working Memory (VWM) indices for the DMR eye-tracking task. Secondly, we proceed with the correlation analysis to assess the role of perinatal characteristics within each group of participants for each tool. Lastly, we performed a correlation analysis across all measurements and participants so that we explore the relationship among the perinatal characteristics, Bayley-III and DMR eye-tracking irrespective of prematurity.

## 3 Results

### 3.1 Developmental assessment data

The infants’ development was evaluated via the Greek adaptation of the Bayley III. Table 2 summarizes the descriptive statistics per group with regard to the Cognitive, Language and Motor Scales.

**Table 2:** Bayley-III: mean index score per group.

	Preterm infants	Full term infants
Cognitive Scale	93.7 ± 2.2	95.4 ± 2.05
Language Scale	102.5 ± 3.04	104 ± 3.02
Motor Scale	86.8 ± 5.3	89 ± 10.01

In order to analyze the Bayley-III data, we performed analysis of variance (one-way ANOVA) to compare the effect of Prematurity on Cognitive, Language and Motor indices. The analysis revealed no statistically significant differences between preterm and full-term infants suggesting no developmental differences between the two groups as it is measured via the Bayley-III assessment tool.

Next, we assessed the role of sex, BW, SGA, GA, CA and Severity Index in the performance of preterm and full-term infants. As a first step to our analysis we conducted a Pearson correlation assessing the relationship among Bayley-III measures and BW, GA, CA and Severity Index so as to identify the parameters that appear to boost development in each group. Table 3 summarizes the significant correlation data for preterm infants and Table 4 the significant correlation data for full term infants.

**Table 3:** Pearson correlation data of perinatal characteristics and Bayley-III for preterm 8- to 9-month-old infants (N: 39; * indicates *p* < 0.05, ** indicates *p* < 0.001).

	BW	GA	CA	Severity index	Cognitive scale	Language scale	Motor scale
BW		0.833**	−0.110	−0.839**	0.317	0.165	0.223
GA			−0.138	−0.827**	0.360*	0.239	0.183
CA				−0.160	−0.209	−0.268	−0.119
Severity index					−0.367*	−0.167	−0.278
Cognitive scale						0.650**	0.553**
Language scale							0.612**
Motor scale							

**Table 4:** Pearson correlation data of perinatal characteristics and Bayley-III for full term 8- to 9-month-old infants (N: 24; * indicates *p* < 0.05, ** indicates *p* < 0.001).

	BW	GA	CA	Cognitive scale	Language scale	Motor scale
BW		0.389	0.032	0.310	−0.163	0.114
GA			−0.265	0.373	0.428*	0.174
CA				−0.203	−0.305	0.000
Cognitive Scale					0.249	0.573*
Language Scale						0.388
Motor Scale						

The preterm data (Table 3) reveal some expected strong correlations between BW and GA, and GA and Severity Index. Interestingly, GA and Severity Index also correlate with the Cognitive scale of Bayley-III but not with the other two scales. The Cognitive, Language and Motor scales though do correlate positively and strongly suggesting that the three domains move in the same pace for preterm infants. The full-term data (Table 4), on the other hand, do not show extended correlations among perinatal characteristics and developmental scores apart from two cases; interestingly, GA appears to correlate positively with the Language Scale, and secondly, Cognitive Scale and Motor Scales appear to correlate strongly and positively to each other.

With regard to any possible effects of sex, the analysis of variance did not reveal any differences between female and male preterm infants but there were some differences with regard to full term infants. Specifically, male full term infants had a smaller GA [*F*_(1, 23)_ = 4.668, *p* = 0.042, *d* = 0.906, Male: 38.3 < Female: 39.7] and scored lower in the Cognitive Scale [*F*_(1, 23)_ = 5.1994, *p* = 0.033, *d* = 1.113, Male: 92.1 < Female: 102.8]. Additionally, with regard to the preterm infants, we tested the role of SGA; the analysis showed an expected drop in BW for SGA preterm infants [*F*_(1, 36)_ = 7.629, *p* = 0.009, *d* = 0.980, SGA: 1457 < Non-SGA: 2073] along with a significant decrease in performance with regard to the Cognitive Scale of Bayley-III [*F*_(1, 36)_ = 4.021, *p* = 0.054, *d* = 0.813, SGA: 85.5 < Non-SGA: 97]. The FGR status did not appear to have an effect on the performance of preterm infants.

Overall, the analysis of the Bayley scores showed that this developmental assessment tool is not sensitive to prematurity since it does not discriminate the two CA-matched groups, preterm and full-term infants participating in the study. Yet, it allows us to record the role of GA in cognitive development and some individual differences relating to perinatal characteristics in the dataset.

### 3.2 DMR eye-tracking data

Turning now to the DMR eye-tracking task, we extracted fixation time data developing two types of measurements: Visual Attention (VA) and Visual Working Memory (VWM) indices. With regard to Visual Attention, we assessed (a) the sum of overall attendance during the task, (b) the total fixation time on all three AOIs, and (c) the fixation time on the object. In reference to Visual Working Memory data, we measured the gaze time on object-location bindings in the match and mismatch pair conditions. The descriptive statistics per group are summarized in Table 5.

**Table 5:** DMR eye-tracking data – visual attention and visual working memory indices.

		Preterm infants	Full term infants
VA	Overall attendance [%]	39.88 ± 16.87	52.13 ± 14.8
	Total AOIs	25.08 ± 17.41	37.42 ± 15.85
	Object Fixation T	23.61 ± 16.61	33.88 ± 17.12
VWM – object location bindings	Match	1.67 ± 1.72	2.72 ± 2.12
	Mismatch	1.26 ± 1.02	2.26 ± 2.47

To evaluate differences among infants, we performed analysis of variance with Prematurity as the independent variable (preterm vs. full term infants) and the Visual Attention and Visual Working Memory Indices as the dependent variables. The statistical analysis showed that full term infants outperformed preterm infants across all measurements; Overall attendance: [*F*_(1, 61)_ = 8.566, *p* = 0.005, *d* = 0.771], Total AOIs: [*F*_(1, 61)_ = 7.972, *p* = 0.006, *d* = 0.741], Object Fixation T: [*F*_(1, 61)_ = 5.555, *p* = 0.022, *d* = 0.608], Match object location bindings: [*F*_(1, 61)_ = 4.653, *p* = 0.035, *d* = 0.543], Mismatch object location bindings: [*F*_(1, 61)_ = 5.086, *p* = 0.028, *d* = 0.529]. The gaze data revealed a main effect of Prematurity with significant delays in the development both of visual attention and visual working memory as measured via the DMR eye-tracking task.

Next, similarly to the Bayley-III analysis, we proceed with the examination of the role of sex, BW, SGA, GA, CA and Severity Index in the performance of preterm and full-term infants in the DMR eye-tracking task. As a first step to our analysis we conducted a Pearson correlation assessing the relationship among the Visual Attention and the Visual Working Memory Indices and BW, GA, CA and Severity Index so as to identify the parameters that appear to boost development in each group. Tables 6, 7 summarize the significant correlation data for preterm infants and full-term infants, respectively.

**Table 6:** Pearson correlation data of perinatal characteristics and DMR eye-tracking measures for preterm 8- to 9-month-old infants (N: 39; * indicates *p* < 0.05, ** indicates *p* < 0.001).

	VA – Overall attendance	VA – Total AOIs	VA – Object Fixation T	VWM – Match object location bindings	VWM – Mismatch object
BW	0.249	0.065	0.031	−0.135	0.124
GA	0.346*	0.192	0.166	−0.063	0.066
CA	0.132	0.050	0.038	0.330*	0.269
Severity Index	−0.252	−0.044	−0.019	0.064	−0.127
VA – Overall attendance		0.891**	0.872**	0.567**	0.292
VA – Total AOIs			0.992**	0.495**	0.232
VA – Object Fixation T				0.475*	0.153
VWM – Match object location bindings					0.633**
VWM – Mismatch object location bindings					

**Table 7:** Pearson correlation data of perinatal characteristics and DMR eye-tracking task for full term 8 to 9 month old infants (N: 24; *indicates *p* < 0.05, ** indicates *p* < 0.001).

	VA – Overall attendance	VA – Total AOIs	VA – Object Fixation T	VWM – Match object location bindings	VWM – Mismatch object
BW	0.129	0.041	0.063	−0.193	−0.011
GA	0.526*	0.563*	0.545*	−0.338	−0.233
CA	−0.217	−0.173	−0.159	0.257	0.178
VA – Overall attendance		0.859**	0.854**	−0.159	−0.327
VA – Total AOIs			0.970**	−0.076	−0.242
VA – Object Fixation T				−0.236	−0.435*
VWM – Match object location bindings					0.782**
VWM – Mismatch object location bindings					

The correlation analysis of the preterm data (Table 6) showed that GA correlated positively with visual attention while CA correlated with visual working memory skills as it was indicated by the Overall attendance and Match object location binding indices, respectively. Meanwhile, the severity index did not appear to hinder the performance of preterms in the DMR task. Note though that both VA and VWM indices correlated strongly and positively with each other suggesting that their development is aligned. Turning now to the full-term data (Table 7), the analysis shows that GA correlated positively and strongly with all three measurements of visual attention, and VA and VWM indices correlated to each other. Note also that for full term infants more gaze time on the object led to less gaze time in the mismatch location suggesting better function of VWM which is not attested in the preterm dataset.

With regard to any possible effects of sex, the analysis of variance did not reveal any differences between female and male preterm infants but there was a difference with regard to full term infants. Significant longer gaze time by female full term infants is recorded in reference to the VA measurement of Total AOIs [*F*_(1, 23)_ = 4.626, *p* = 0.047, *d* = 0.899, Male: 33.3 < Female: 47.3]. Furthermore, we examined the role of SGA in preterm infants; the analysis showed a change in performance with regard to Object Fixation T [*F*_(1, 36)_ = 3.807, *p* = 0.059, *d* = 0.627, SGA: 30.70 < Non-SGA: 19.57] and the Match object location bindings indices [*F*_(1, 36)_ = 5.371, *p* = 0.026, *d* = 0.718, SGA: 2.61 < Non-SGA: 1.22]. Moreover, FGR preterm infants showed performance effects in the Match object location bindings measurement [*F*_(1, 36)_ = 3.874, *p* = 0.057, *d* = 0.622, FGR: 2.44 < Non-FGR: 1.30]. Lastly, we explored whether there are any significant correlations between the eye-tracking and Bayley measurements for preterm and full-term infants, but no such correlation was identified in each of the datasets. Overall, the results suggest that prematurity and GA in particular are critical factors in VA and VWM measurements and the DMR eye-tracking task is a sensitive enough tool to assess the development of infants that may be going unnoticed by other type of measurements.

### 3.3 Re-evaluating perinatal characteristics past prematurity

The analysis so far has demonstrated that the Bayley-III does not discriminate preterm to full term infants, while attention and working memory measurement extracted via the DMR eye-tracking task successfully identified the differences between the two groups. Moreover, some diverse patterns were shown within each dataset in relation to their perinatal characteristics; BW, GA and CA appear to be the most primary of features across the two populations we tested suggesting that they can be significant tools when exploring neurodevelopment during the first year of life. Consequently, as a final step in our analysis, we evaluate the role of BW, GA and CA on Bayley-III and DMR tasks across participants conducting a Pearson correlation in order to assess the relationship among Bayley-III and DMR eye-tracking measures and BW, GA and CA and potentially identify the characteristic that appears to have the greatest explanatory value developmentally across infants. Table 8 summarizes the significant correlation data of those variables.

**Table 8:** Pearson correlation data of perinatal characteristics, Bayley-III and DMR eye-tracking measures across 8- to 9-month-old infants (N: 63; *indicates *p* < 0.05, ** indicates *p* < 0.001).

	BW	GA	CA	Bayley-III			DMR Eye-tracking				
				Cognitive scale	Language scale	Motor scale	VA – Overall attendance	VA – Total AOIs	VA – Object Fixation T	VWM – Match object location bindings	VWM – Mismatch object
BW		0.874**	−0.160	0.269*	0.092	0.212	0.267*	0.253*	0.205	0.077	0.229
GA			−0.260*	0.286*	0.225	0.175	0.491**	0.412**	0.364*	0.112	0.175
CA				−0.204	−0.184	−0.081	−0.088	−0.114	−0.111	0.218	0.128
Cognitive Scale					0.531**	0.560**	−0.020	0.000	−0.012	−0.133	−0.061
Language Scale						0.545**	0.091	0.060	0.073	−0.098	−0.082
Motor Scale							0.016	0.031	0.033	−0.058	−0.067
VA – Overall attendance								0.894**	0.875**	0.343*	0.053
VA – Total AOIs									0.983**	0.327*	0.063
VA – Object Fixation T										0.230*	−0.089
VWM – Match object location bindings											0.715**
VWM – Mismatch object location bindings											

The correlation analysis of the full dataset (Table 8) showed that BW positively correlates with the Cognitive Scale of Bayley and two of the VA measurements of the eye-tracking task, specifically, Overall attendance and Total AOIs. Similarly, the GA of infants also positively correlates with the Cognitive Scale of Bayley (more strongly so) and all three of the VA measurements of the eye-tracking task. Note that there are significant correlations for GA, BW and CA, which is expected. Another important finding is that even when looking across infants there is no correlation between Bayley-III and DMR measurements suggesting that as tools there is no overlap in the areas of development and the particular cognition domains they assess. Yet, within each task there are strong correlations among their indices.

## 4 Discussion

The present study set out to explore the discriminatory value of eye-tracking methodology in comparison to off-line testing in preterm and full-term infants with the aim to identify possible neurodevelopmental delays within the first year of life so that early intervention can be introduced. The analysis revealed that the DMR eye-tracking task via visual attention and visual working memory measurements identifies significant differences due to prematurity suggesting that there is great potential in eye-tracking methodology in reliably tracking neurodevelopmental delays in infancy. Meanwhile, the developmental assessment tool Bayley III does not appear to be sensitive to prematurity since it does not discriminate the preterm and full-term 8- to 9-month-old infants of the study.

Prematurity is associated with many neonatal complications (Patel, 2016; Humberg et al., 2020; Smyrni et al., 2021) that may result in cognitive delays and developmental disorders in childhood (Buck et al., 2000; Magny and Rigourd, 2003; Jarjour, 2015; Pascal et al., 2018; Lowe et al., 2020; Beunders et al., 2021 among others). In addition, perinatal characteristics often linked to prematurity, such as BW, GA, SGA and FGR, appear to affect brain development and cognitive skills such as learning and memory and as such they are considered to be high risk factors for neurodevelopmental difficulties (see Amin et al., 2013; Bickle Graz et al., 2015; Meher et al., 2015; Sacchi et al., 2020; Larsen et al., 2022; Naz et al., 2023). The perinatal period and the first year of life are key in neurodevelopmental terms and a number of ERP studies have identified impairments that related to the development of the brain and of specific areas such as the hippocampus (deRegnier et al., 2000; Siddappa et al., 2004; Geng et al., 2015). Neurodevelopment is typically monitored via the clinical assessment and the administration of standardized tests such as the Bayley Scales (Bayley – III, 2006b; Yue et al., 2019; Del Rosario et al., 2021).

The administration of the Bayley Scales has indeed assisted in the early detection of developmental delays in preterm infants (Aylward, 2002), however, most studies have reported cognitive, language, and motor neurodevelopmental difficulties due to prematurity at the CA of 2 to 2;6 years (Duncan et al., 2012) or at the CA of 12 months (Velikos et al., 2015) without any information on whether the Bayley Scales could detect delays earlier on within the first year of life. Moreover, performance in such standardized tests has not been explored in relation to other type of measurements such as eye-tracking methodologies. Considering this gap in the literature, we attempted to explore how informative the administration of Bayley Scales is in relation to prematurity. In addition, we developed a novel assessment tool, a Delayed Match Retrieval (DMR) paradigm via eye-tracking, which measures visual working memory and attention skills. We examined its ability to discriminate between preterm and full term infants at the CA of 8 to 9 months providing the first dataset in the literature on such a young age – for data on children tested via eye-tracking at the age of 1 and 2 years see Beunders et al. (2021). It is important to note that the Bayley Scales is a norm-referenced assessment tool of overall early childhood development, while the DMR eye-tracking task we developed exploits visual attention and working memory skills that are a part of cognition. As such, the eye-tracking task assesses a particular cognitive domain, which the Bayley Scales may only partly tap on.

Earlier studies on gaze data have employed varied stimuli including motion stimuli, cartoon and audio stimuli to engage the infant’s attention (see Kaldy et al., 2016; Kaul et al., 2016 among others). The task design of Kaldy et al. (2016), for example, was that of a memory game with three face-down virtual cards, two flipped over sequentially (e.g., a card with a swirl pattern, a card with a star), and then flipped back, while the third card was flipped to reveal a match to one of the previously presented cards. Meanwhile, Kaul et al. (2016) used an optoelectronic camera system to assess eye and head movements while the infant tracked a moving object. Moreover, earlier work suggests that infants older than 8 months appear to have the capacity to remember about three objects (Kibbe and Leslie, 2013), yet the uniqueness of the objects may have an effect on the processing capacity of the infants (Oakes et al., 2013). Considering these findings, the DMR eye-tracking task we developed employs the standardized LEA symbols in black and white, which are of similar processing complexity so as to minimize any competition effects in how infants responded and possibly masking their actual performance in the task. Additionally, the ability of infants to respond to stimuli that are more complex appears to increase past the age of 7 months (Swingler et al., 2015) and there is research evidence from diverse cultural backgrounds suggesting that visual objects can operate as units of attention at the age of 8 months (Bulf and Valenza, 2013; Tsurumi et al., 2018).

In view of these earlier findings, we opted for testing 8- to 9-month-old infants. Specifically, we administered the DMR task to 63 preterm and full-term infants along with Bayley-III. Even though the number of participants was relatively small, the analysis revealed significant differences between the two groups. Starting with the Bayley data, the analysis showed that this assessment tool is not sensitive enough to prematurity since it did not discriminate between the preterm and full term 8- to 9-month-old infants of the study. Meanwhile, the DMR eye-tracking data clearly discriminated between the two groups across all measurements tapping on both Visual Attention (VA) and Visual Working Memory (VWM) suggesting significant delays in cognitive development due to prematurity.

Starting with the VA indices, our data is in line with the findings of the Kaul et al. (2016), Ross-Sheehy et al. (2017) and Beunders et al. (2021) studies who related visual attentional deficits to prematurity even though there were variations with regard to the ages they tested. Turning to the VWM indices, our data offers support to the Kaldy et al. (2016) study which tested a limited number of full-term infants, specifically fourteen 8-month-old and twelve 10-month-old infants using a similar eye-tracking experiment. Similarly to Kaldy et al. (2016), we identified the value of eye-tracking methodology in tracking cognition measuring working memory skills. Yet our study tested a much more extended data pool and added the diverse effects related to prematurity and perinatal characteristics, which were not addressed by Kaldy et al. (2016) (for object-location bindings in full term 8-month-old typically developing infants see Bulf and Valenza, 2013; Tsurumi et al., 2018; for discussion on development of alerting, orienting, and executive attention see Oakes, 2023). The Beunders et al. (2021) cohort study, on the other hand, explored an extensive dataset of 1 and 2 year old children using cartoon, motion and form stimuli. Despite the differences in their experimental design in terms of stimuli design and age of testing compared to the present study, their findings are in line with our study’s outcomes; that is that nonverbal eye-tracking tasks can detect adverse cognitive development.

The correlation analysis of the preterm data additionally showed that GA correlated with visual attention while CA correlated with visual working memory skills; yet it is important to note that more gaze time on the object led to less gaze time in the mismatch location in the full-term dataset suggesting better function of VWM which was not found in the preterm dataset. Moreover, the preterm dataset did not reveal any sex related effects, while SGA and FGR preterm infants’ performance dropped significantly offering further support to earlier studies that identify SGA and FGR status relating to higher risk for cognitive issues (Algarín et al., 2003; Atkinson and Braddick, 2007; Fransson et al., 2007; Amin et al., 2013; Bickle Graz et al., 2015; Meher et al., 2015; Larsen et al., 2022; Naz et al., 2023 among others). Overall, the results suggest that prematurity and GA in particular are critical factors in VA and VWM measurements and the DMR eye-tracking task is a sensitive enough tool to assess the development of preterm infants that may be going unnoticed by other screening tools.

Next, we explored possible correlations across all participants. The correlation analysis showed that BW and GA positively correlate with the Cognitive Scale of Bayley and the VA measurements of the eye-tracking DMR task. Note also that both when looking within the preterm and full-term datasets and when looking across all infants of the study there was no correlation between Bayley-III and DMR measurements suggesting that as tools there is no overlap in the areas of cognitive development they assess. The possible implications of this result are the following (a) the two tools assess different areas of cognition, (b) their data require different interpretation due to the fact that the Bayley-III provides off-line measurements while the eye-tracking DMR task provides on-line measurements, and (c) broad assessment tools may not be able to identify the developmental milestones of executive functions within the first year of life while a focused test may be able to track individual differences and can provide a quantifiable measurement. Nevertheless, both tools provide valuable information and within each task there are strong correlations among their indices. Thus, the cognitive delays reported in our eye-tracking dataset verifies that cognitive impairment is relating to preterm birth and perinatal features relating to prematurity (van de Weijer-Bergsma et al., 2008; Mulder et al., 2009; Atkinson and Braddick, 2012; Lowe et al., 2020; Beunders et al., 2021 among others). Our findings overall suggest that depending on the tool employed developmental delays due to prematurity may be missed; hence, future research and clinical practice would benefit by the addition of eye-tracking tasks that monitor several domains of neurodevelopment, such as cognition and language.

Nonetheless, the present study has some limitations. Recruiting infants and especially premature ones can be challenging and as such the sample analyzed even though more extensive than most studies it is relatively small. Additionally, there is no follow-up that would allow us to examine their performance longitudinally and provide further support to our research findings. Yet, we managed to test infants at a very young age and provide a comparison between a standardized assessment tool and a novel eye-tracking task, which is one of the main contributions of our study and a future step for us is to retest those participants at a later stage. Given the evidence for associations between foundational executive functional skills and later cognitive and academic ability, future research is required to focus on extending the datasets currently available via adding more eye-tracking evidence on various stages within the first year of life so that we offer support and intervention as early as possible in the vulnerable group of premature infants.

To conclude, visual working memory and attention as measured via the DMR eye-tracking task is a non-intrusive, painless, short duration procedure (approx. 4-min) that was found to be a significant tool for identifying prematurity and FGR effects on the development of cognition even at the very young age of 8 months, while Bayley Scales alone would not pick up these deficits. Prematurity is an issue affecting the community, it is linked to short- and long-term neonatal complications and it is associated to greater risk for cognitive difficulties in childhood. Consequently, identifying tools for early neurodevelopmental assessments and cognitive function is important in order to enable earlier support and intervention in the vulnerable group of premature infants.

## Data availability statement

The raw data supporting the conclusions of this article will be made available by the authors, without undue reservation.

## Ethics statement

The studies involving humans were approved by Scientific Council and the Ethics Committee of Papageorgiou General Hospital of Thessaloniki, Greece (Ethics Approval N: Δ3β/28893|D3b/28893). The studies were conducted in accordance with the local legislation and institutional requirements. Written informed consent for participation in this study was provided by the participants’ legal guardians/next of kin.

## Author contributions

MK: Writing – review & editing, Writing – original draft, Validation, Resources, Methodology, Investigation, Formal Analysis, Data curation, Conceptualization. EB: Conceptualization, Data curation, Writing – review & editing, Writing – original draft, Resources, Methodology, Investigation. GF: Writing – review & editing, Writing – original draft, Methodology, Investigation, Formal Analysis. EG: Writing – review & editing, Writing – original draft, Investigation. KK: Writing – review & editing, Writing – original draft. KN: Writing – review & editing, Writing – original draft, Conceptualization. VS: Writing – review & editing, Writing – original draft, Project administration, Methodology, Conceptualization.

## Footnotes

^1^For ease of read we use CA for full term infants, with the only difference being that CA and chronological age coincide for full term participants.

^2^To ensure the calmness and cooperation of infants we piloted the experiment with infants siting at their caregiver’s lap or at a children’s highchair that provided support to the infants’ head with no presence of the adult caregiver. Infants appeared to benefit when sitting in their caregiver’s lap and this set-up was selected for the administration of the task.

^3^During piloting, we also tested the administration of the two sets with a few minutes break in between and it led to drop in commitment.
